# Basic and Translational Advances in Glioblastoma

**DOI:** 10.1155/2018/1820345

**Published:** 2018-03-26

**Authors:** Sara G. M. Piccirillo, Marta M. Alonso, Francesco Pasqualetti

**Affiliations:** ^1^UT Southwestern Medical Center, Dallas, TX, USA; ^2^University Hospital of Navarra, Pamplona, Spain; ^3^Pisa University Hospital, Pisa, Italy

Glioblastoma (GBM) is the most aggressive brain tumor in adults. Due to the high degree of heterogeneity, little is known about the molecular events underpinning GBM genesis and the disease is already advanced at clinical presentation, thus complicating the efficacy of therapies.

This special issue provides an update on novel basic and translational advances, which are expected to impact on patient treatment and improve survival.

On the translational front, the review by F. A. Chowdhury et al. focuses on the cytotoxic and antiapoptotic potential of Thymoquinone for GBM treatment by affecting key signaling pathways that promote tumor cell proliferation. Another two reviews by V. Desai and A. Bhushan and M. N. Park et al. highlight the potential of natural compounds in the treatment of this devastating disease both individually and in combination with the standard of care for GBM patients, that is, chemo- and radiotherapy, and their mechanism of action in promoting apoptosis and suppressing invasion. Another novel therapeutic strategy based on recombinant immunotoxins is described by S. Zhu et al. in their review. Importantly, this work also highlights the challenges associated with the use of immunotoxins, for instance, delivery to the brain and immunogenicity.

Finally, the original papers by C.-N. Im and by D. Kavaliauskaitė et al. propose two experimental strategies to inhibit GBM growth. One is based on PPAR*γ* inhibition using its ligands and its inhibitor that results in downregulation of stem cell markers and sphere-forming ability and the other on sodium valproate that acts by blocking the proliferation, migration, and angiogenesis of human glioma cells.

As part of the basic advances in GBM research, we solicited the submission of manuscripts that could help deepen our understanding of the signaling pathways underlying malignant growth and ultimately lead to the development of new therapies. The research article by S. Paglia et al. focuses on alterations in the PTEN/aPKC/Lgl axis that are implicated in gliomagenesis in* Drosophila*. The review by L. Ryskalin et al. addresses the role of the mammalian Target of Rapamycin (mTOR) in the brain and in particular how mTOR activation is critical in GBM development. H. Alfardus et al. examine the key role of microRNA in the regulation of glycolytic metabolism and oncogenic signaling pathways in GBM. Finally, a review by S. A. Valdés-Rives et al. provides an overview of all the signaling pathways that negatively control apoptosis and how these could be targeted to trigger an apoptotic response in GBM.

In an elegant and alternative strategy, using mathematical algorithms M.-E. Oraiopoulou et al. modelize not only overall proliferation but also the intratumor heterogeneity. Moreover, these in silico data are further validated using* in vitro* models. Another interesting approach for screening of different antiglioma compounds is shown by D. W. Lee et al. This group implemented a 3D cell-based, high-throughput screening method that allows mimicking the microenvironment using a micropillar and microwell chip platform. Using this system, they evaluate cytotoxicity and efficacy of 70 compounds.

In line with the notion that the microenvironment plays an important role in sustaining the pathogenesis of glioma, I. A. W. Ho and W. S. N. Shim investigated the contribution of the microenvironment to GBM heterogeneity. In their work they try to understand how the different types of stromal cells that surround the tumor such as endothelial cells, microglia, and others may impact angiogenesis, invasion, proliferation, and stemness of GBM cells. Understanding this crosstalk would aid to better tailor therapeutic interventions.

Finally yet importantly, the work by N. Senhaji et al. characterizes the EGFR amplification and IDH mutations in GBM patients from the Northeast of Morocco. In this work the authors, using a specific population, aim to determine the prevalence of those genetic aberrations and how this relates to the frequency seen in other ethnic groups.

We hope that this special issue will help investigators interested in GBM biology to familiarize with the recent therapeutic advances in this field and to better understand the role of key signaling pathways in the oncogenic process leading to this disease. We also hope that the content of this special issue will stimulate discussion and inspire future basic and translational studies.

*Sara G. M. Piccirillo* *Marta M. Alonso* *Francesco Pasqualetti*

